# Monitoring Perinatal Health in Europe: Strengths and Challenges of the Euro‐Peristat Project

**DOI:** 10.1111/ppe.70024

**Published:** 2025-04-20

**Authors:** Thillagavathie Pillay

**Affiliations:** ^1^ Faculty of Science and Engineering University of Wolverhampton Wolverhampton UK; ^2^ Neonatal Services, Women and Children's Directorate University Hospitals Leicester NHS Trust Leicester UK; ^3^ Department of Population Health Sciences University of Leicester Leicester UK

Maternal health influences perinatal outcomes (viable foetus and neonatal), including survival, and contributes to health throughout an individual's lifetime [1]. Optimising these remains a global health challenge. In Europe, maternal mortality and stillbirth rates have stalled or slowed in the last decade [2], although neonatal mortality continues to decline [3]. That mortality has superdeterminants overlaying its clinical picture [4], including disparities in the social drivers of health [5], health systems, and resources, suggests that multi‐faceted approaches are required to progress and sustain improvements in maternal and perinatal health in the region and globally.

The utility of large‐scale real‐world epidemiological data to explore variation between regions is a valuable tool enabling benchmarking and identifying population trends to drive research, quality improvement, health service policy, and delivery changes. These are described in Figure 1. While aspirational, understanding variation in this context cannot be done purely through randomised controlled trials in perinatal health. This is mainly due to the unpredictability of the pregnancy course and its outcomes, the need for large cohorts, and the inability to offer representation across all population groups and European socio‐economic strata [6].

**FIGURE 1:**
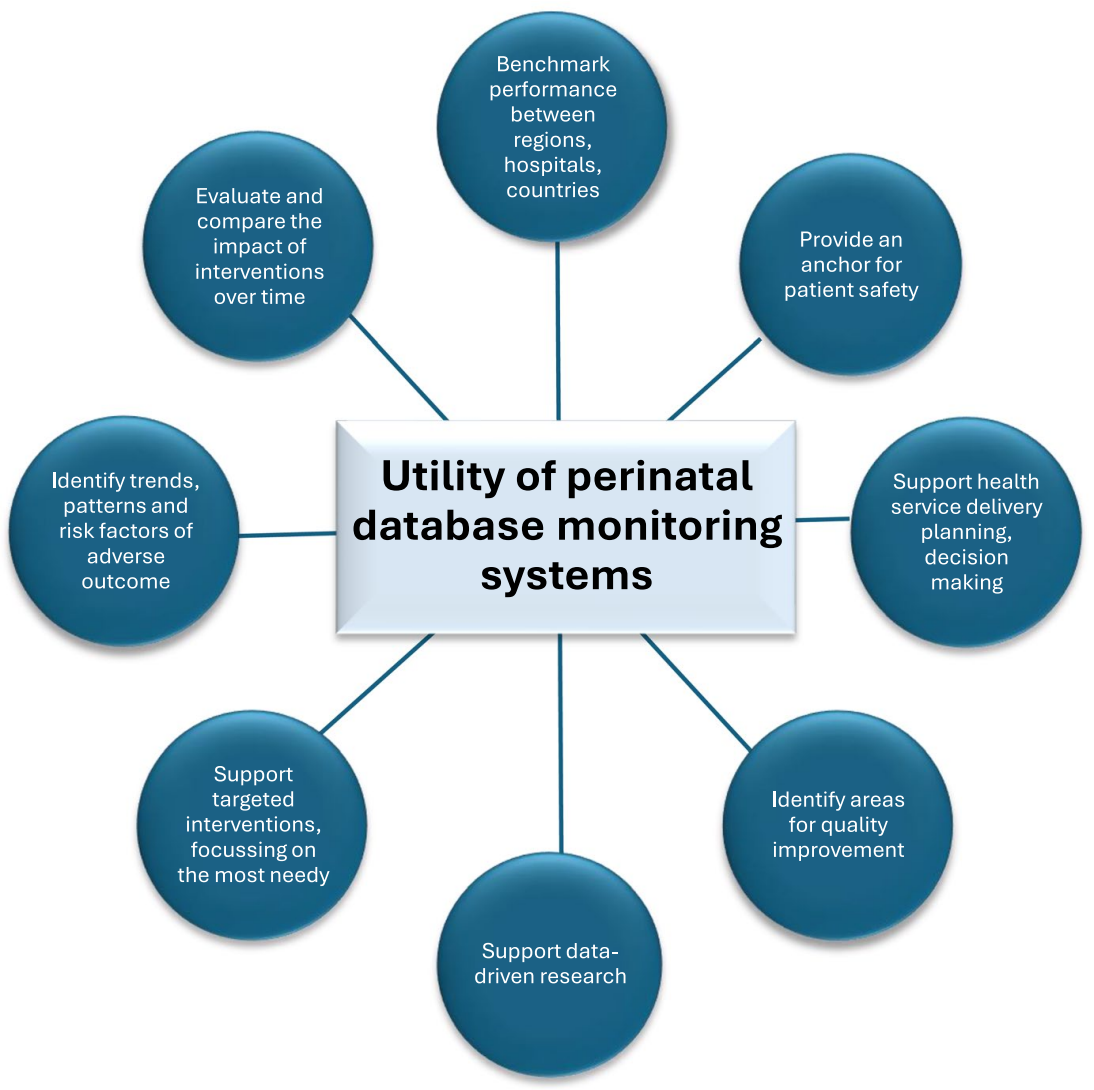
Potential utility of perinatal database monitoring systems.

In this issue of *Paediatric and Perinatal Epidemiology* [7], the team from Euro‐Peristat outlines available data sources in 29 European countries that provide data on core perinatal health indicators. Established almost a quarter of a century ago as part of a European Union health monitoring programme, their report outlines substantial progress, but lays bare the challenges in the development of a central European data model for maternal and perinatal outcomes.

So, what are some of these challenges? To be effective, core outcomes captured in robust databases that are, in the first instance, nationally inclusive, complete, and then internationally comparable with interoperability are essential. Currently, there is high diversity in the collation of multiple core outcomes [7]. National healthcare policies vary based on societal expectations and perspectives. As such, there is variation in definitions of stillbirth, what is classified as a livebirth, at which gestational age life‐sustaining support is offered, and whether there is an opt‐in versus opt‐out policy for delivery of care at the extremes of viability, including congenital anomalies.

Standardising record keeping also varies across European nations. For example, capturing outcomes for citizens versus residents, recording termination of pregnancies, and home versus hospital births. Illegal migrant data are also not routinely captured, and worryingly, perinatal health databases may not be representative of the most vulnerable, underserved communities across Europe, which are at the highest risk for adverse perinatal outcomes. There may be multiple sources of linkage, and data linkage to mortality is not standard practice for all countries. In Philibert et al.'s description [7], only 16 countries linked birth to death data, which had to be implemented as part of the Euro‐Peristat programme. Availability of health service resources may mean that the calibre of data entry, apart from the quality of care offered, will vary between countries, and regions within countries. Data validation and correction require intense work—in the Euro‐Peristat context, only a third of 29 out of 31 participating countries had complete data. These challenges are not restricted to the Euro‐Peristat [8].

However, comparator analysis between nations may allow us to examine (i) whether variations in outcome reflect variation in policy and distribution of resources, (ii) whether practices in one country are transferable to another, with similar effect, and (iii) whether socio‐economic determinants of health outcomes could be modifiable in the same way between nations and between generations. These questions may not be answerable by a database [5], but they provide a robust, rich contextual source against which individual countries and regions can be benchmarked.

Currently, the key parameter being harmonised is survival. However, other more subtle outcomes must also be considered when monitoring perinatal health. Preterm birth and a range of maternal and neonatal conditions fit the bill [9], but these are again limited by the complexities of pooling together large‐scale routinely collected data and determining common themes for monitoring. Capturing outcomes on third‐degree perineal tears, mode of delivery, preterm birth, and low birthweight in regional contexts is a start. Understanding the impact of maternal risks and confounders in perinatal health outcomes is essential, too.

The value of large‐scale real‐world databases in serving as a helpful resource that drives optimisations of health service delivery is straightforward [7, 10]. We now need to understand how better to ensure that the data is representative, standardised where necessary, and validated as a must. Real‐time outputs that can influence data‐driven exploration and research, resulting in timely identification, monitoring, change and review are aspirational. Euro‐Peristat is a step in the right direction.

## Author Contributions

T.P. conceptualised and drafted the commentary.

## Disclosure

The comments and views of the author are not necessarily those of the University of Wolverhampton or University Hospitals Leicester NHS Trust, UK.

## Conflicts of Interest

The author declares no conflicts of interest.

## Data Availability

The author has nothing to report.
